# Editorial: Stromal and immune cell interactions in intestinal inflammation and fibrosis

**DOI:** 10.3389/fimmu.2023.1152140

**Published:** 2023-02-16

**Authors:** Vassilis Valatas, Kazuya Kitamura, Stephen G. Ward, George Kolios

**Affiliations:** ^1^ Department of Gastroenterology, University Hospital of Heraklion, Heraklion, Greece; ^2^ Department of Gastroenterology, Tonami General Hospital, Tonami, Japan; ^3^ Department of Life Sciences, University of Bath, Bath, United Kingdom; ^4^ Faculty of Medicine, Democritus University of Thrace, Alexandroupolis, Greece

**Keywords:** intestinal stromal cells, mesenchymal cells, inflammatory bowel disease (IBD), IL-34, extracellular matrice (ECM), glycolysis (glycolytic pathway), intestinal fibrosis, cuproptosis

During the last decade accumulating data demonstrated that intestinal stromal cells are not just a supporting structure that responds homogenously to immune stimuli. Rather they represent a complex organization of mesenchymal cells, an organ within the intestine, with discrete roles for every one of its members, orchestrating complex tasks. Unfortunately, full characterization of stromal cell subsets and comprehension of their discrete functions has been hampered by the absence of a standardized nomenclature. This is mostly attributed to species differences and the variable experimental approaches used for characterization as murine mechanistic studies are based on surface markers of isolated cells, whereas human descriptive studies are based mostly on single cell transcriptomics.

In the current topic, Chalkidi et al. and Sun et al. align together many pieces of the puzzle in two comprehensive reviews that examine murine and human intestinal mesenchymal subsets in parallel and summarize their molecular phenotypes and functions during intestinal homeostasis and inflammation. CD81^+^ fibroblasts, (also called trophocytes) in the small intestine, type III stromal cell in the human colon or crypt bottom fibroblasts, are located below crypts. They are analogous to human *WNT2B* ^+^ cells and their main function is to maintain intestinal stem cell identity and proliferation *via* antagonizing BMP signal while promoting the Wnt signal by expressing *Grem1*, *Wnt2*, *Wnt2b*, *Wnt5a,Rspo1* and *Rspo3* (1, 2). PDGFRα^hi^ fibroblasts, also called Pdgfra^hi^Foxl1^+^ murine telocytes or CD142^+^ human telocyte-like cells, are equivalent to human *PDGFRA* ^+^, *WNT5B* ^+^, S2 subsets. They contain two distinct subsets that are located at the top and bottom of villi and crypts, and regulate epithelial cell differentiation, proliferation, and maturation *via* enhanced expression of Wnt and BMP ligands (1, 3). PDGFRα^lo^CD81^-^ murine intestinal fibroblasts or stromal 1 cells in humans, are located in the lamina propria, around crypts and inside the villous core. Analogous to human *WNT2B* ^+^ *FOS* ^+^ lamina propria fibroblasts, they secrete basement membrane proteins and contribute to extracellular matrix (ECM) production, remodeling and intestinal stem cell niche formation (1), as well as maintenance of lacteal integrity (4).

These distinct intestinal mesenchymal stromal cells and their subclasses seem to organize sub-epithelial immune regulatory centres that can interact with different types of immune cells as reviewed by Sun et al. In brief, stromal cells support innate defenses through the production of cytokines such as IL-6, and chemokines that recruit neutrophils (IL-8), monocytes (CCL-2) and T cells (CCL-5) to promote bacterial clearance (5). Organized in discrete immune hubs at the villus tip and the crypt bottom, stromal cells enhance innate immune responses or regulate autoimmunity respectively. This is mediated through co-operation with specific innate lymphoid cell populations and various immunoregulatory mechanisms that include expression of PD-L1 and PD-L2 immune checkpoint inhibitors  (Beswick et al.), regulation of retinoid acid in tolerogenic dendritic cells (6) and expansion of regulatory *FOXP3^pos^ T cells* (7).

During inflammation, stromal cells both produce and respond to cytokines and chemokines through a variety of cytokine receptors that can be up-regulated by inflammatory stimuli (8, 9). In response to the inflammatory milieu of human Inflammatory Bowel Disease (IBD) or murine experimental IBD, stromal cells broaden their chemokine production (10–12) and have been implicated in treatment-resistant disease in IBD patients that overproduce Oncostatin M or IL-1β (13). Furthermore, they produce factors involved in fibrosis like IL-11, which is also part of the IL-6 family, and TNFSF15 (14; West). IBD is also associated with expansion of fibroblast clusters with high expression of PDPN or FAP and up-regulation of inflammation associated genes such as CCL-19, IL-33, TNFSF14, IL-1R1, TNFSF11, and IL-13RA_2_ (10, 12) in parallel with diminished capacity of intestinal fibroblasts to induce IL-10-producing regulatory T cells (7). The differential expression of PD-L1 in the two main clinical entities of IBD on intestinal stromal cells; i.e. Crohn’s disease (CD), and Ulcerative colitis (UC) with a decreased PD-L1 expression in patients with UC, but an increased PD-L1 expression in CD patients may indicate an involvement in the observed distinct immunopathological changes characteristically seen in these two entities (Beswick et al.).

In the current topic, Monteleone et al., summarize data on production of IL-34 and M-CSF-1 by intestinal stromal cells that regulates resident macrophages to promote gut homeostasis. In the context of intestinal inflammation, blockage of M-CSF1 or IL-34 or loss of M-CSF-1R protects mice in many experimental colitis settings. A parallel pro-fibrotic role of IL-34 in human CD is demonstrated by its higher expression in fibrostenotic samples and its ability to up-regulate ECM production by human intestinal stromal cells. This underscores the presence of CD34-driven inflammatory and fibrotic amplification loops in the intestinal mucosa that may represent a new therapeutic anti-inflammatory and anti-fibrotic target in IBD.

Intestinal stromal cell derived ECM has been shown to participate in engagement of immune cells during inflammation, in profibrotic cascades or in proper wound healing after inflammation has subsided (15). However, Seltana et al. report that fibrinogen produced by a subset of intestinal epithelial cells has a primary role in proper wound healing. They show that epithelial derived fibrinogen is deposited at the basement membrane as fibrin where it serves as a substrate for wound healing under physiological conditions. During inflammation, inhibition of fibrin formation exacerbates experimental dextran sulfate sodium (DSS) colitis indicating that epithelial-derived fibrin is important for preventing epithelial damage or promoting epithelial repair. This highlights important interactions between stromal and epithelial cells during the intestinal wound healing responses.

Previous studies have shown that glycolysis is the preferred energy source for fibroblasts in fibrosis, while inhibition of glycolysis decreases fibrosis by reducing ECM (16, 17). The metabolic enzyme 6-phosphofructo-2-kinase/fructose-2, 6-bisphosphatase 3 (PFKFB3) is a key kinase regulating glycolytic activity and it is implicated in inflammation and various immune-related disorders (18). In the current issue, Zhou et al., present data for the role of PFKFB3 in patients with IBD. Using single-sample gene set enrichment analysis, they found that glycolysis is significantly higher in human intestinal samples from IBD patients compared to healthy controls, while single-cell sequencing data showed that PFKFB3 expression is higher in gut biopsies from IBD patients. Furthermore, PFKFB3 expressed in the stromal cells of the intestinal samples is upregulated following stimulation with proinflammatory cytokines in *in vitro* models. In addition, inhibition of PFKFB3 by the specific inhibitor PFK15 results in reversion of the increased expression of pro-inflammatory cytokines and chemokines in fibroblasts and reduction of the severity of DSS and T cell transfer induced colitis. This study demonstrates for first time that increased PFKFB3 expression in stromal cells contributes to inflammation and fibrosis in IBD and suggests that inhibition of PFKFB3 might be a new therapeutic strategy in intestinal inflammation and fibrosis.

In the context of recurrent autoinflammatory intestinal disorders as in IBD, post-inflammatory intestinal fibrosis may occur that alters mesenchymal cell biologic behavior. Intestinal fibroblasts reduce migration, and increase proliferation and collagen production, in parallel clusters that regulate epithelial cell proliferation are diminished (12, 19–22). In this issue, Dovrolis et al. approach fibrosis as a universal link in disease. By identifying common fibrotic transcriptomic signatures, in otherwise uncorrelated disorders, and their site-specific co-expression, they proceed to apply this knowledge on the issue of ileal fibrosis during IBD. Their methodology provides the basis for a practice that can be applied similarly to other shared molecular mechanisms. Meanwhile, their findings illuminate the molecular background of a serious complication in IBD, opening the way for future treatment-focused mechanistic studies or applications in other fibrotic disorders.

Finally, in the current Research Topic, Chen et al., giving attention to a new research field, have deeply examined the expression of cuproptosis-related regulators and their implication in the pathogenetic mechanisms of IBD. Recently, a copper-dependent pathway that differs from all other known pathways that underlie cell death, called cuproptosis, has been described. Cuproptosis occurs *via* copper binding to lipoylated enzymes in the tricarboxylic acid cycle, which leads to proteotoxic stress and cell death, and it has been reported to be involved in various disorders. In the present study, Chen et al. apply bioinformatic strategies to examine for first time, the implication of cuproptosis in IBD. They examine the association between immune score and cuproptosis-related genes, and they present a comprehensive landscape of their importance in IBD. This study therefore identifies cuproptosis as playing a key role in the pathogenesis of IBD with its ability to change chronic death of the intestinal epithelial cells that might be involved in both disease risk and treatment.

Collectively, these publications in the current Research Topic highlight the role of stromal cells in the pathogenesis of intestinal inflammation and fibrosis. They have focused on a wide range of characteristics of these cells, from their involvement in inflammatory mechanisms and their crosstalk with immune cells and epithelial cells of the intestinal mucosa, through soluble mediators, to their pivotal role in the fibrotic process of IBD. Furthermore, the Research Topic examines the heterogeneity of stromal cell subtypes and their contribution to epithelial repair and immune homeostasis in gut mucosa, giving these cells a primary role in mucosal immunology.

## Author contributions

VV, KK, SW and GK edited the Research Topic and wrote this editorial. All authors approved the submitted version of this editorial.
